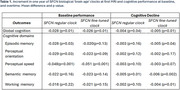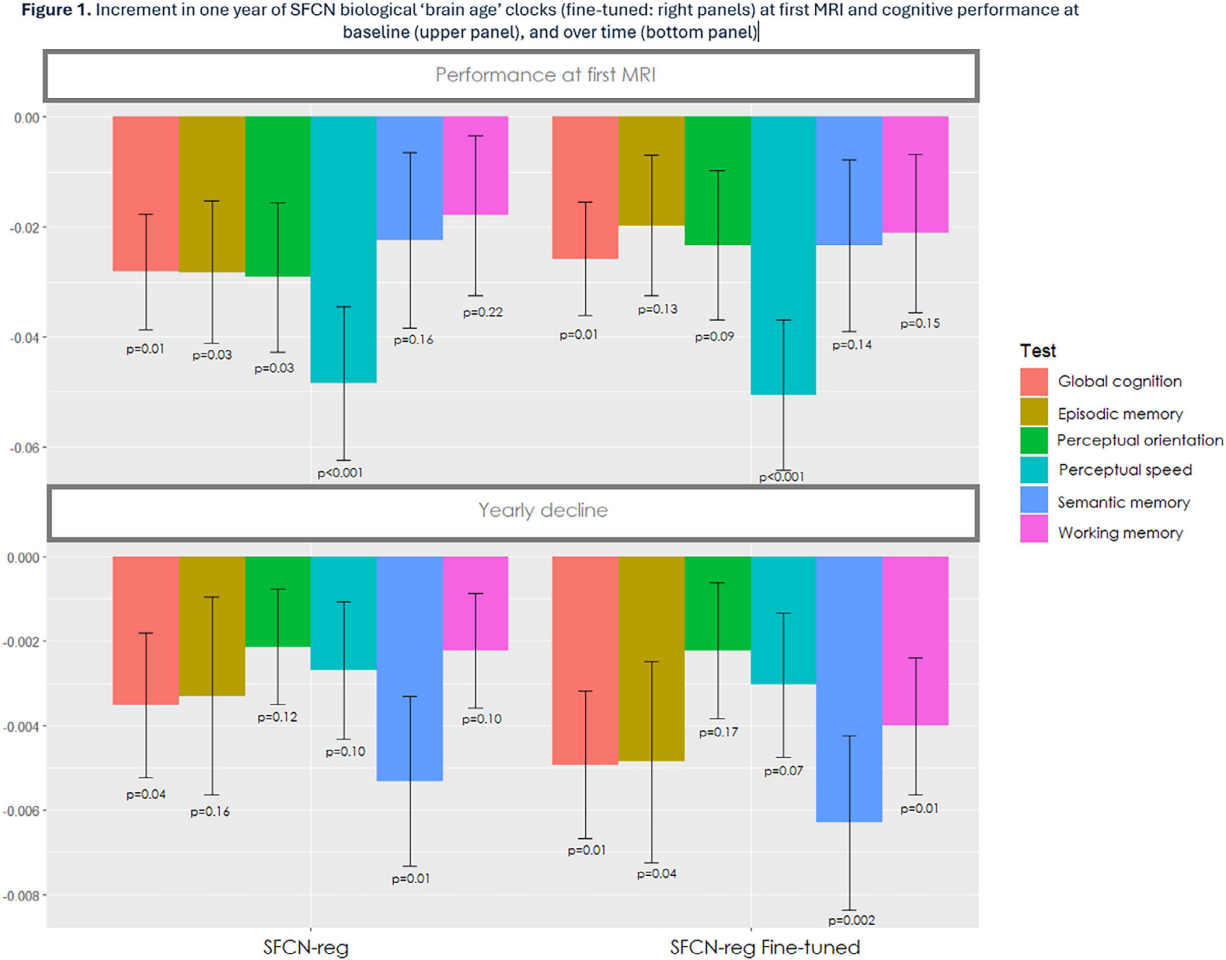# MRI‐derived biologic clocks and cognitive aging in a sample of older Latino adults

**DOI:** 10.1002/alz70856_101389

**Published:** 2025-12-25

**Authors:** Jocelyn Jaen, Konstantinos Arfanakis, Yingjuan Wu, Melissa Lamar, Francine Grodstein

**Affiliations:** ^1^ Rush Alzheimer's Disease Center, Chicago, IL, USA; ^2^ Department of Diagnostic Radiology and Nuclear Medicine, Rush UniversityRush University Medical Center, Chicago, IL, USA; ^3^ Rush University Medical Center, Chicago, IL, USA; ^4^ Department of Psychiatry and Behavioral Sciences, Rush University Medical Center, Chicago, IL, USA; ^5^ Department of Internal Medicine, Rush University Medical Center, Chicago, IL, USA

## Abstract

**Background:**

MRI‐derived ‘brain age’ clocks are promising biomarkers of brain health; however, their application across diverse populations is limited. Evaluating existing MRI‐derived clock(s) in more diverse populations is crucial until algorithms using more diverse populations are developed. We calculated the Simple Fully Convolutional Networks regression variant (SFCN‐reg) MRI‐derived biological clock using structural neuroimaging data of Latinos, given Latinos’ high rates of neurologic diseases like stroke and dementia.

**Method:**

Self‐identified Latinos of Rush Alzheimer's Disease Center (RADC) cohort studies contributed 3T neuroimaging and cognitive data (*N* = 125; mean age=74y; mean education=12y; 82% female). T1‐weighted data were used to estimate SFCN clocks at the first‐valid 3T MRI. In addition to the SFCN‐reg clock, we also calculated a fine‐tuned version revising algorithmic item weights based on data from the older RADC neuroimaging cohort (including Latinos). Longitudinal cognitive data from 19 tests starting from the first valid 3T MRI were used to create annual global and five domain‐specific composite scores. Unadjusted Pearsons Product Moment Correlations evaluated correlations between SFCN‐reg clocks and chronological age. Linear mixed effects models estimated associations between SFCN‐reg clocks (separately) and each cognitive composite score adjusting for confounders including scanner.

**Result:**

Latinos’ mean SFCN‐reg brain age was 69 (SD 6.4) years, while the fine‐tuned SFCN‐reg version was 74 (SD 6.2) years. Correlations between chronological age and original and fine‐tuned SFCN‐reg brain ages were 0.73 and 0.70, respectively. Each one‐year increment in SFCN‐reg brain age corresponded to a 0.028‐unit reduction in baseline global cognitive scores (*p* = 0.01) for the regular clock, and a 0.026‐unit reduction for the fine‐tuned clock (Table 1). Distinct associations between each SFCN‐reg clock and baseline domain‐specific cognition were identified (Figure 1). Both clocks were associated with faster decline in global cognition over time. For domain‐specific cognitive decline, the SFCN‐reg clock did not reach statistical significance in most domains, yet its estimates’ direction and magnitude were similar to those of the fine‐tuned clock.

**Conclusion:**

In this sample of older Latinos, SFCN‐reg clocks correlated with chronological age and cognition, performing well in our Latino cohort. Research with larger Latino samples could further demonstrate SFCM‐reg clocks as promising biomarkers of brain health in this group.